# Clinical characteristics of pneumonia patients of long course of illness infected with SARS-CoV-2

**DOI:** 10.1515/med-2022-0465

**Published:** 2022-05-18

**Authors:** Wang Chunli, Huang Liya, Lu Weiwei, Chen Guoxi, Cai Yuyang, Li Xiaopan, Lan Xing, Wang Yaling, Deng Xiaoqin, Zeng Guangwang, Wang Lin, Ji Chen, Huang Hai, Yang Ling

**Affiliations:** Department of Geriatrics, Xinhua Hospital Affiliated to Shanghai Jiao Tong University School of Medicine, Shanghai, China; Department of Tuberculosis ward 2, Wuhan Pulmonary Hospital, Wuhan, Hubei, China; School of Public Health, Shanghai Jiao Tong University School of Medicine, Shanghai, 200025, China; Center for Disease Control and Prevention, Pudong New Area, Shanghai 200136, China; Fudan University Pudong Institute of Preventive Medicine, Pudong New Area, Shanghai 200136, China; The Health Center of Nansheng Town, Wuzhishan, Hainan Province, China; Department of Clinical Trials Unit, Warwick Medical School, Coventry, England; Department of Tuberculosis ward 2, Wuhan Pulmonary Hospital, Wuhan 430030, Hubei province, China

**Keywords:** pneumonia, long courses, COVID-19, SARS-CoV-2

## Abstract

Epidemiological and clinical characteristics of patients with COVID-19 have been reported in the last two years. A few studies reported clinical course of illness of median 22 days, including viral shedding of median 20 days, but there are several cases with a longer time of viral shedding. In this study, we included four cases with a longer illness course of more than 40 days who had been discharged or still in hospital by March 15, 2020. Demographic, clinical treatment, and laboratory data, including serial samples for viral RNA detection, were extracted from electronic medical records. We described the epidemiological and clinical characteristics and the course of viral shedding. Two patients had comorbidity, one with hypertension and the other with diabetes. We found smoking was not an independent risk factor. D-dimer maybe related to the severity of illness but not to the course of the illness. Nucleic acid detection suggested that maybe more sampling sites represented more virus replication sites and longer course of illness. In this study we found some non-critical severe relatively young patients whose character was different from former studies described to provide a basis for reference to assess the risk of transmission and the isolation duration of patients.

## 1 Introduction

In December 2019, a novel atypical pneumonia caused by the 2019 novel coronavirus (2019-nCoV) was reported in Wuhan, China [1]. As of March 15, 2020, there were 80,860 confirmed cases in China, including 3,213 deaths reported by Chinese government. Now an outbreak of 2019-nCoV all over the world is reported. It not only caused huge psychological trauma to thousands of families but brought huge economic burden for all throughout the world. Most pathogenic coronaviruses only caused mild clinical symptoms in humans [2], except severe acute respiratory syndrome coronavirus (SARS-CoV) [3] and Middle East respiratory syndrome coronavirus (MERS-CoV) [4]. The 2019-nCoV pathogen is confirmed to be closely related to SARS-CoV [5].

Homology modeling revealed that 2019-nCoV had a receptor-binding domain structure similar to that of SARS-CoV, 2019-nCoV might use ACE2 as the receptor [6], and it binds ACE2 with higher affinity than SARS-CoV [7]. There are some amino acid mutations in the 2019-nCoV receptor-binding domain. Studies also suggested that high expression of ACE2 may increase susceptibility to infection [8]. The 2019-nCoV showed interesting new phenomena, for example, there were some asymptomatic infections. Some patients discharged from hospital were tested positive again by real-time RT-PCR assay of nasal and pharyngeal swab specimens from the upper respiratory tract. In addition, some patients with long courses of illness were continually non-negative with acid test. Some scholars suggest that it is a chronic infectious disease and the virus may coexist with humans like HIV and HBV.

Previous study showed the median duration of viral shedding was 20 days (interquartile range [IQR] 17–24) with the longest duration being 37 days. The duration varied for patients with different health conditions, in critical and severe patients, it was 24 days (IQR 22–30) and 19 days (IQR 17–22), respectively, and even shorter in moderate patients [9]. In addition, long duration of disease/viral shedding is usually observed in patients with older age or underlying medical conditions [9]. But in this study, we found the opposite, herein we report four young patients who were no more than 50 years old, with a good health condition before, having a long viral shedding of more than 40 days. The study is aimed to provide a basis for reference to assess the risk of transmission and the isolation duration of patients.

## 2 Materials and methods

### 2.1 Study design and participants

All patients in the study are from Wuhan Pulmonary Hospital, Wuhan, Hubei province, China. The date of admission to hospital is from January 6 to February 17, 2020, and the observation deadline is up to March 15, 2020. We collected four patients no more than 50 years old, with a good health condition before, who had a long course of disease/viral shedding for more than 40 days. These patients were diagnosed as having 2019-nCoV pneumonia on the basis of the World Health Organization (WHO) interim guidance [10]. The viral target fragments of 2019-nCoV specific nucleic acid detection technology are viral ORF1ab and N genes.


**Ethics approval and consent to participate:** This study was approved by the Ethics Committee of Xinhua Hospital Affiliated to Shanghai Jiao tong University School of Medicine (approval number: XHEC-D-2020-042), and performed in accordance with the Declaration of Helsinki. Written informed consent was waived by the Ethics Commission of the designated hospital for emerging infectious diseases.

### 2.2 Procedures

We obtained general information, clinical treatment, and outcome data from patients’ medical records. All data were entered and checked by two physicians. The date of the disease onset was defined as the day when the first symptom was noticed. The course of the disease is calculated using the date of obtaining two consecutive negative test results using nucleic acid-based detection minus the date of disease onset. A confirmed case of COVID-19 was defined as a positive result on real-time RT-PCR assay of nasal and pharyngeal swab specimens from the upper respiratory tract. Only laboratory-confirmed cases were included in the analysis. All patients had chest computer tomography (CT) scan.

### 2.3 Outcomes

The primary end point was discharge from hospital.

## 3 Results

### 3.1 Demographic data and clinical symptoms

Case 1 was a 33-year-old women without underlying comorbidities. The patient had no exposure to Huanan Seafood Market, which was linked to the first reported COVID-19 case. She is a non-smoker. Her first symptoms were cough and dizziness. The temperature was no more than 38°C in her later disease course. She was diagnosed as having moderate pneumonia and still in hospitalization at the end of our data collection. Her illness course exceeded 40 days.

Case 2 was a 50-year-old women with hypertension. She is a non-smoker. She had COVID-19 exposure because of her physician occupation. The first symptoms were cough, sore throat, and myalgia. Her body temperature was no more than 38°C all through her illness course. She was diagnosed as having moderate pneumonia, and her illness course was 40 days. She was discharged from hospital on day 40.

Case 3 was a 36-year-old male without underlying comorbidities and a non-smoker. He had confirmed COVID-19 pneumonia patient exposure because of his physician occupation, his first symptoms were cough, fever, and later diarrhea. His highest body temperature was up to nearly 40°C before admission to hospital. He was diagnosed as having severe pneumonia, and his illness course was more than 40 days. He was still in hospital.

Case 4 was a 46-year-old man with diabetes and he was a non-smoker. He had Huanan Seafood Market exposure. His first symptoms were cough and chest tightness, and later shortness of breath. His highest body temperature was 37.7°C before admission to hospital. He was diagnosed as having severe pneumonia, and his illness course was more than 50 days. He was still in hospital.

### 3.2 Nucleic acid tests

**Figure 1:**
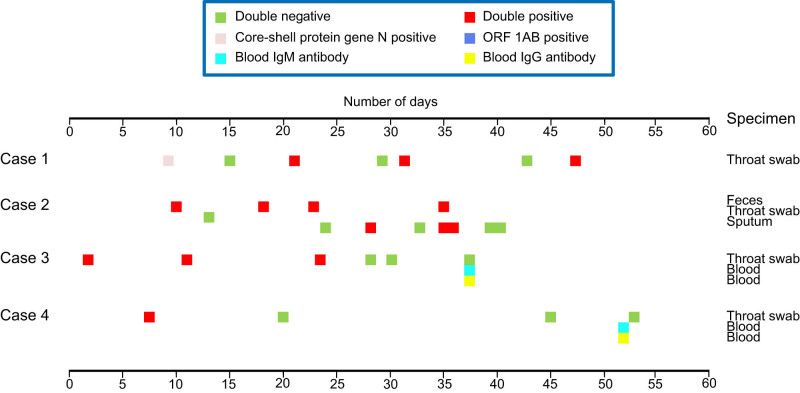
Disease/virus shedding based on nucleic acid detection of four patients.

All nucleic acid test results and the corresponding sampling sites of the four patients are shown in Figure 1. All cases had both double-negative and double-positive result at different times during the disease course. It is worth noting that sampling sites of case 2 were stool, sputum, and throat swab specimens, while the other three cases were sampled from throat swab specimens. Cases 3 and 4 were also tested for blood antibody. Case 2 had two consecutive double-negative results and was discharged from hospital, while other three cases were still in hospital.

### 3.3 Laboratory tests

**Figure 2:**
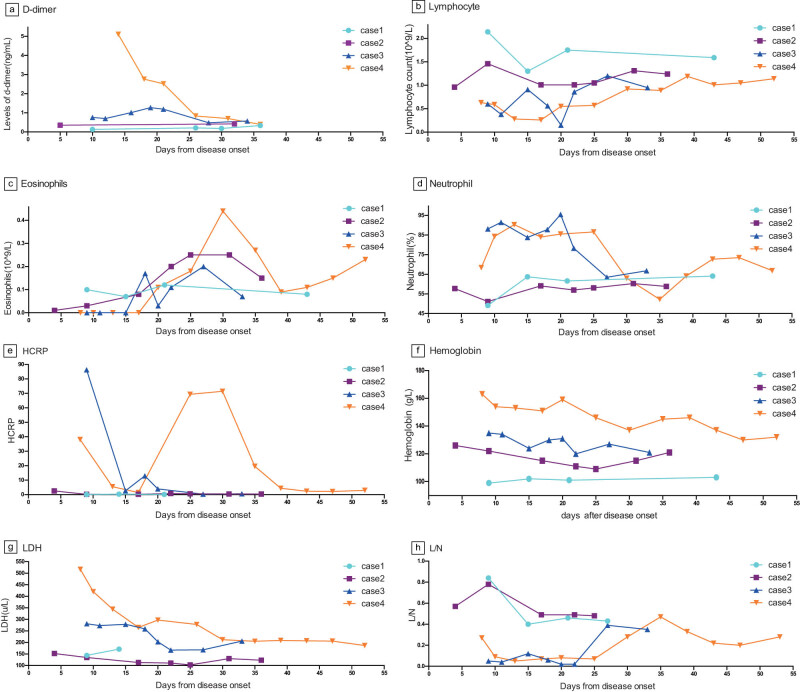
Laboratory values of four patients. (a) Levels of D-dimer of four patients. (b) Lymphocyte count of four patients. (c) Eosinophil count of four patients. (d) Neutrophil (%) of four patients. (e) HCRP of four patients. (f) Hemoglobin of four patients. (g) LDH of four patients. (h) Lymphocyte/neutrophil of four patients.

Laboratory values are shown in Figure 2. Two severe cases (3 and 4) had elevated D-dimer level, while two moderate pneumonia cases (1 and 2) did not. This is consistent with the previous studies that the severity of disease is related to D-dimer level. Cases 2, 3, and 4 had decreased lymphocyte count. Case 3 had the largest reduction in lymphocyte count of 0.15 × 10^9^. This patient also constantly had higher temperatures, whether it suggests that the body temperature is related to lymphocyte count needs further confirmation. All four cases had elevated eosinophil count. It reached the highest level in the days 25–30, followed by a slow decrease, with more fluctuation in cases 3 and 4, first reduced to lower than normal, even zero the lowest in about days 10, and increased slowly, to the highest in the days 25–30, and subsequently decreased slowly. Increased neutrophil occurred in cases 3 and 4. The elevation of HCRP (hypersensitive C-reactive protein, hs-CRP) was also observed in cases 3 and 4. These two cases had secondary bacterial infection following virus infection. Maybe it suggests that the secondary bacterial infection has an influence on the severity of the disease, but if secondary bacterial infection exists it has no relation to the course of the illness. The two severe cases had no anemia, while the two moderate cases were observed to have anemia from the hemoglobin data. It suggested that the decreased hemoglobin has no relation to the course of illness. Elevated lactate dehydrogenase (LDH) was found in the two severe cases, while decreased lactate dehydrogenase was found in case 2. It suggests that maybe the elevated LDH is related to the severity of the disease, but had no effect on the course of illness. Lymphocyte/Neutrophil data is shown to be in the lower level in first 2–3 weeks, and returned normal slowly after 3 weeks.

### 3.4 Imaging examinations

**Figure 3:**
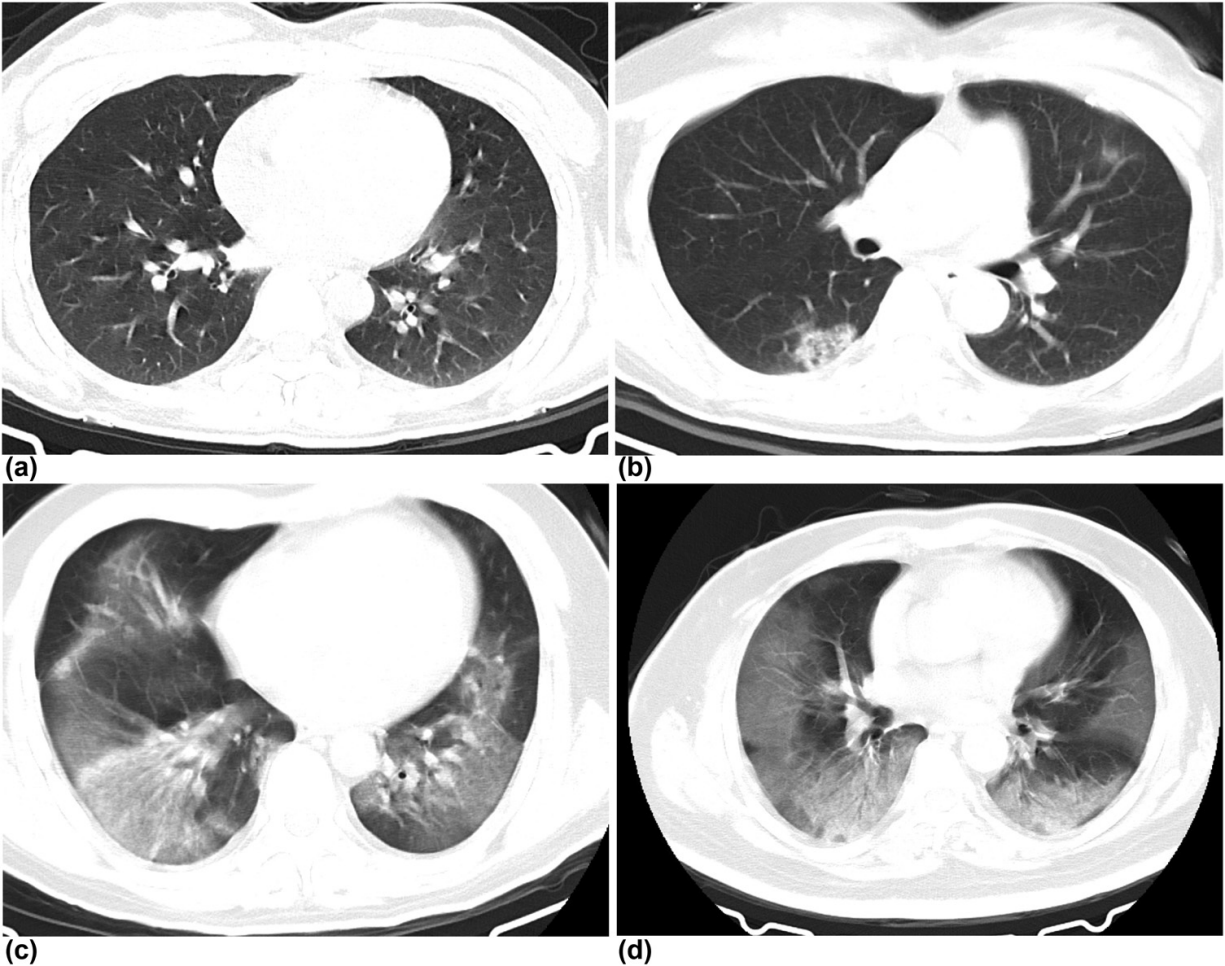
Chest CT images of four patients. (a) Transverse chest CT image from case 1 showing slight ground-glass opacity in the left lower lobe area on day 5 after symptom onset. (b) Transverse chest CT image from case 2 showing bilateral multiple patchy shadows with uneven density and unclear borders on day 7 after symptom onset. (c) Transverse chest CT image from case 3 showing bilateral multiple large diffuse ground-glass opacity on day 11 after symptom onset. (d) Transverse chest CT image from case 4 showing bilateral multiple large diffuse ground-glass opacity with obvious outer lateral distribution on day 11 after symptom onset.

Intact CT imaging of all cases are available. Features of CT imaging, including location, proportion, and number of associated lung lobes and manifestation, are shown in the supplementary table. Ground-glass opacity in both the lungs were characteristically observed on CT image in all cases except case 1. Peripheral distribution was observed in case 1, and bilateral sides involvement were observed in other three patients. Pleural effusion and thickened pleura were not detected in any of the cases (Figure 3).

### 3.5 Treatment

**Figure 4:**
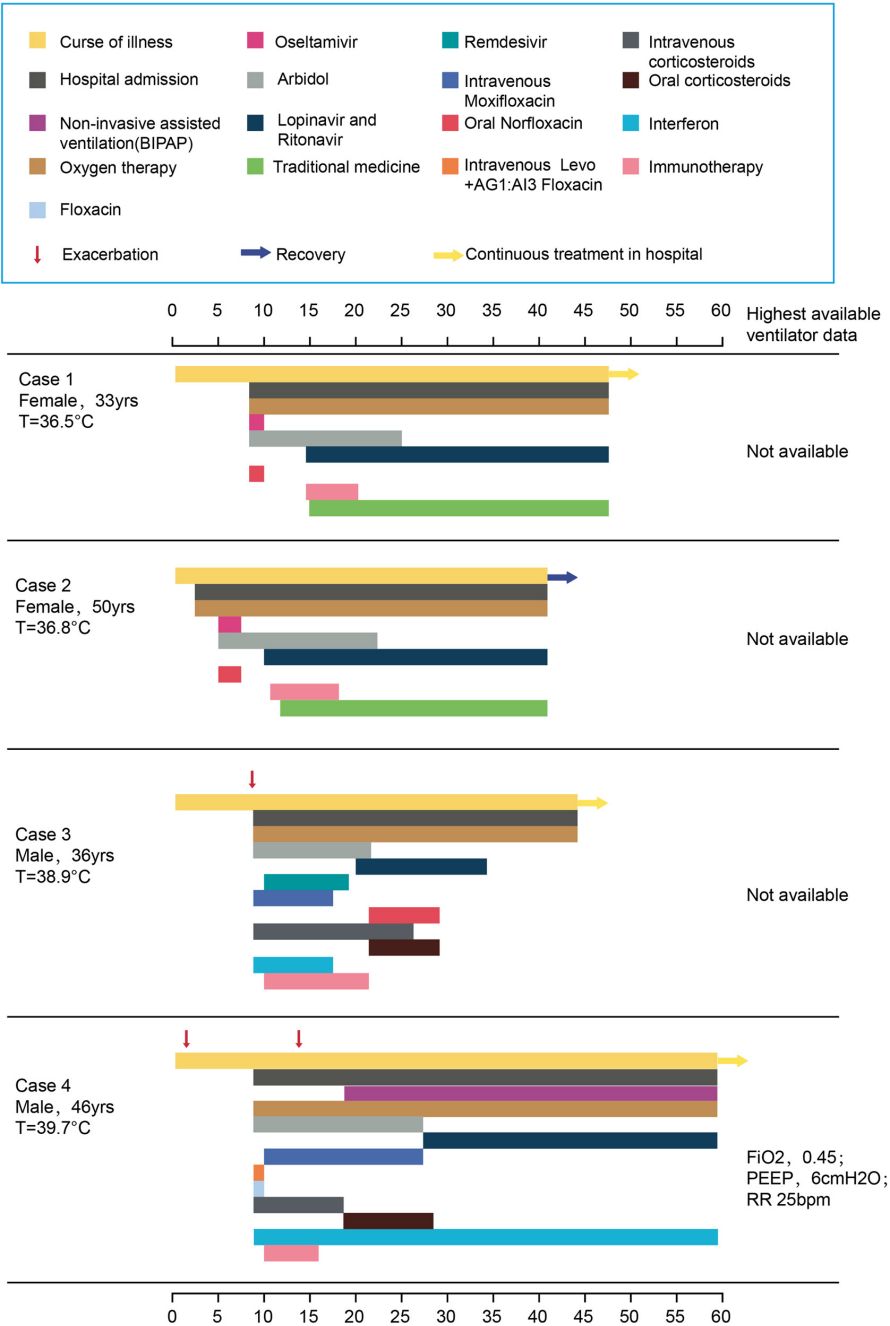
Clinical course and treatment (FIO_2_ denotes fraction of inspired oxygen, PEEP positive end-expiratory pressure).

Antiviral drugs, mechanical ventilation, immunotherapy, and oxygen therapy were administered to all patients. Intravenous antibiotics and corticosteroids were given to two severe patients (Figure 4). Traditional medicine (Novel coronavirus pneumonia 3 decoction) was given to two patients having moderate pneumonia. During the course of the illness, case 4 was given two antiviral drugs: Arbidol and Lopinavir-Ritonavir Tablets, two antibiotics: moxifloxacin and Levo + AG1:AI3 floxacin, 18 days of corticosteroids in all. Elevated ALT level was observed only in case 4. Case 2 was given three antiviral drugs: Arbidol, Lopinavir-Ritonavir, and Oseltamivir, and Chinese traditional medicine. And only case 2 had drug caused leukopenia. Virus infection is a self-limited disease, although many things ofthe novel COVID-19 pneumonia is not clear, if it suggests the over-treatment of these patients needs consideration.

### 3.6 Complications and outcome

Until now, only case 2 was discharged from hospital after two consecutive double-negative results. The other three cases were still in the hospital. The two severe cases had hypoalbuminemia, and case 4 had respiratory failure. All four cases had no exacerbation.

## 4 Discussion

2019-nCoV is a novel coronavirus that can be detected in the gastrointestinal tract, saliva, and urine, but there are many things that are still unclear, including how the virus is transmitted from person to person, whether the virus can be spread by the fecal-oral route, how does the virus replicate in different sites and whether that is related to the severity of the disease? If nucleic acid based detection exists false-positive or false-negative, how much is the rate of the false-positive or negative of nucleic acid detection, and why some patients are sustained non-negative with nucleic acid based detection.

Previous research confirmed older patients with comorbidities seem to be easily developing severe disease, and the detectable 2019-nCoV RNA persisted for median 20 days, prolonged virus shedding was associated with fatal outcome [9]. In this study we find several young patients no more than 50 years old, and having a better basic physical condition, to be infected with the disease. While their prolonged virus detection was more than 40 days, the disease type was moderate or severe, and all of them survived. We found the doctor and the nurse of the four patients had clear contact with the confirmed patients. As a typical RNA virus, the average evolutionary rate for coronaviruses is roughly 10^−4^ nucleotide substitutions per site per year, with mutations arising during every replication cycle [2], and if this suggests that there are different mutations coexisting in the same patient, this needs deep observation and sequence of detection.

Although it is reported that the number of deaths associated with 2019-nCoV have a lower fatal rate than either SARS-CoV or MERS-CoV, the transmission is much more rapid than SARS-CoV or MERS-CoV [11,12]. The duration of infectious virus replication is important to assess the risk of transmission and the isolation duration of patients. To ease the tension of hospital and health workers, research needs to be carried out on the non-severe young patients with long course of illness. Previous studies confirmed the detectable 2019-nCoV persisted for a median of 20 days in survivors, but in this study, we found that in some survivors the detectable 2019-nCoV persisted for more than 40 days. Some scholars suggest 2019-nCoV is a chronic disease like HBV, but from the current evidence and discharged patients, the results are not sufficient to confirm that 2019-nCoV is a chronic illness like HBV. We tend to accept the view of self-limiting acute infectious disease. As to the longer course of illness, further information and study are required.

Based on the expression of ACE2 in smoking individuals, it is inferred that long-term smoking might be a risk factor for 2019-nCoV [13], in this study we showed the evidence against that finding. Therefore, the association between smoking history and COVID-19 might require further investigation.

From the data of nucleic acid detection, we observed that one patient may be double-positive with nucleic acid detection from different secretions. It suggests that more sampling sites represent more virus replication sites and longer course of illness. It also needs further study.

## 5 Conclusion

In conclusion, in this study we found several younger patients who were non-critical severe, with sustained non-negative with nucleic acid detection. It will provide strategy for isolation of infected patients and optimal antiviral interventions in the future.

To provide information quickly, we first introduce four representative patients of long course of illness in this study, and later we will provide more cases of long course. The virus contested was not quantitative, so we cannot confirm if the course of illness is associated with the virus concentration.
